# Unusual gastric mucosal infiltration by a medullary thyroid carcinoma: a case report

**DOI:** 10.1186/s13256-016-0981-9

**Published:** 2016-07-27

**Authors:** T. Karrasch, W. Doppl, F. C. Roller, A. Schäffler, R. Schäffer, S. Gattenlöhner

**Affiliations:** 1 Department of Internal Medicine III, Giessen University Hospital, Klinikstrasse 33, 35392 Gießen, Germany; 2 Central Interdisciplinary Endoscopy Unit (ZIVE), Giessen University Hospital, 35392 Gießen, Germany; 3 Department of Radiology, Giessen University Hospital, 35392 Gießen, Germany; 4 Department of Pathology, Giessen University Hospital, 35392 Gießen, Germany

**Keywords:** Epigastric pain, Medullary thyroid carcinoma, Distant metastasis, Gastric mucosa, Tyrosine kinase inhibitors, Case report

## Abstract

**Background:**

Medullary thyroid carcinoma accounts for approximately 1 to 2 % of all thyroid carcinoma cases. The most common route of dissemination is to locoregional lymph nodes. Distant metastases commonly affect bones, lungs, and liver. We present a case of a white woman with a 25-year history of medullary thyroid carcinoma on multiple medications including tyrosine kinase inhibitor therapy for the last 11 months, who exhibited unusual diffuse infiltration of advanced stage medullary thyroid carcinoma to her gastric mucosa.

**Case presentation:**

A 53-year-old white woman presented with increasing fatigue, loss of appetite, and severe epigastric pain radiating to her back. She had a history of medullary thyroid carcinoma (pT2pN1b), diagnosed 25 years ago and treated by complete thyroidectomy and repeated bilateral cervical lymph node dissection. Medical therapy included octreotide 20 mg every 4 weeks, which was switched to the tyrosine kinase inhibitor vandetanib 300 mg/day 11 months ago when computed tomography scanning revealed progressive mediastinal lymph node and diffuse and symptomatic pulmonary metastases. Of note, she demonstrated macroscopically stable pulmonary and mediastinal lymph node metastases; however, her calcitonin serum levels dramatically increased. Computed tomography scanning revealed a single new intrahepatic lesion (4 mm) as well as multiple (>10) new supraclavicular lesions suggestive of medullary thyroid carcinoma progress. As proven by gastric biopsy and immunohistochemical evaluation, her epigastric pain was explained by a diffuse infiltration of her gastric mucosa by metastatic medullary thyroid carcinoma. Subsequently, she rapidly deteriorated and died.

**Conclusions:**

The current case report shows for the first time an unusual metastatic infiltration of the gastric mucosa by medullary thyroid carcinoma. When treating these patients, it is important to include this differential diagnosis during follow-up.

**Electronic supplementary material:**

The online version of this article (doi:10.1186/s13256-016-0981-9) contains supplementary material, which is available to authorized users.

## Background

Thyroid cancer overall represents a rare tumor entity, however, the incidence of thyroid cancer has recently been constantly increasing [1, 2]. Within this heterogeneous group of tumors, differentiated thyroid cancer represents the majority (approximately 90 %) of all newly diagnosed thyroid cancer cases [3]. Medullary thyroid carcinoma (MTC) accounts for approximately 1 to 2 % of all thyroid carcinoma cases, and at diagnosis 7 to 23 % of all patients demonstrate metastatic disease [1, 4]. Overall, 10-year disease-specific survival rates average approximately 75 % with a strong dependency on tumor stage (averaging 21 % in stage IV tumors). Metastases are commonly found in the lymph nodes, while distant metastatic disease is found in lungs, bone, and liver, and, more rarely, in brain, skin, and breast [5, 6]. Therapeutically, the recent introduction of tyrosine kinase inhibitor therapy (vandetanib, cabozantinib) has led to a significant improvement of the prognosis in patients with rapidly progressive and advanced stage MTC [7].

## Case presentation

A 53-year-old white woman presented with increasing fatigue, loss of appetite, and severe epigastric pain radiating to her back. She had a history of MTC (pT2pN1b), diagnosed 25 years ago and treated by complete thyroidectomy and bilateral cervical lymph node dissection, followed by repeated surgical resection of lymph node metastases 19 and 12 years ago. No further specific treatment modalities (for example radiotherapy) had been employed. No genetic analysis had been performed for germline or somatic *RET* proto-oncogene mutations, nor for other somatic mutations (*HRAS*, *KRAS*, *NRAS*). On clinical examination, however, there was no indication of other manifestations of multiple endocrine neoplasia type 2 (MEN2), especially no clinical signs of pheochromocytoma. Since data on the *RET* proto-oncogene status have been missing in our patient, no risk category (moderate, high, highest) according to the revised American Thyroid Association Guidelines for the management of MTC [1] had been assigned. However, based on the American Joint Committee on Cancer (AJCC) TNM-Classification, the MTC had been a stage IV A tumor (pT2pN1b) at the time of the initial complete thyroidectomy in our patient. Post complete thyroidectomy, she had hypoparathyroidism, which was treated with calcium and calcitriol. She received levothyroxine replacement therapy. Repeated biochemical and clinical evaluations during the course of the disease did not demonstrate any signs of paraneoplastic adrenocorticotropic hormone (ACTH) and/or corticotropin-releasing hormone (CRH) secretion. Medical therapy of the MTC included octreotide 20 mg every 4 weeks, which was switched to the tyrosine kinase inhibitor vandetanib 300 mg/day 11 month ago when computed tomography (CT) scanning revealed progressive mediastinal lymph node and diffuse and symptomatic pulmonary metastases.

**Fig. 1:**
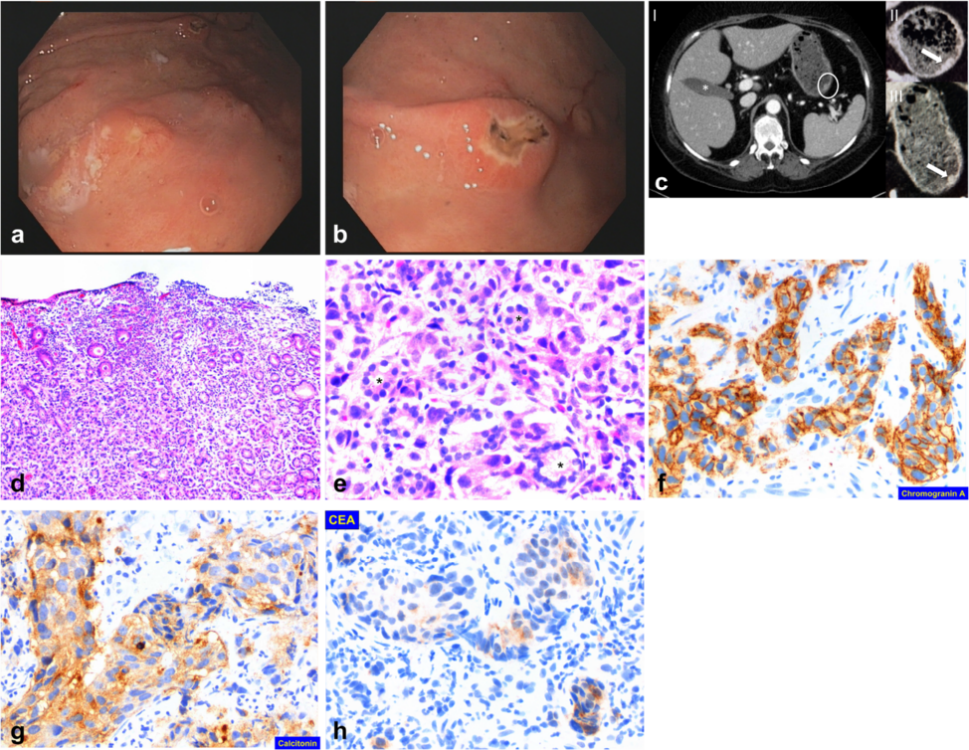
**a**, **b** Endoscopic view of the gastric corpus mucosa. **c** Computed tomography demonstrating multiple small noduli within the stomach wall: I overview, noduli marked by *white circle*; II, III magnification, noduli marked by *white arrows*. **d**–**h** Histologic and immunohistochemical staining of gastric biopsy specimen. **d** Hematoxylin-eosin, overview. **e** Hematoxylin-eosin, detail. **f** Immunohistochemical staining for chromogranin A. **g** Immunohistochemical staining for calcitonin. **h** Immunohistochemical staining for carcinoembryonic antigen (CEA). Remarkably, histology reveals intact non-neoplastic gastric gland structures (marked by *) in between infiltrating cells

Standard laboratory tests in our patient revealed moderate leukocytosis (18.3 G/l) as well as slightly elevated serum CRP levels (12.58 mg/l); her serum lactate was 5.5 mmol/l. Pancreatitis was ruled out because her serum amylase and lipase levels were within normal range. An upper endoscopy was performed and demonstrated multiple centrally ulcerated lesions in her gastric mucosa of up to 4 mm in size (Fig. 1a, b). Two-phase contrast-enhanced multi-detector CT of her chest and abdomen revealed stable pulmonary and mediastinal lymph node metastases; however, there was a single new intrahepatic lesion of 4 mm size suspicious for liver metastasis. Contrast-enhanced multi-detector CT of her neck revealed multiple, new, centrally hypodense supraclavicular lesions up to 12 mm in size suggestive of lymph node metastases. In addition, multiple noduli within her stomach wall were apparent (Fig. 1c, I–III). Three-phase bone scintigraphy showed no evidence of bone metastasis.

Additional laboratory tests revealed a dramatic increase in her basal serum calcitonin levels from 563 pg/ml to 6487 pg/ml within 2 months, indicative of rapid tumor progress of the underlying MTC. Of interest, at the same time she had rather stable carcinoembryonic antigen (CEA) serum levels of 40.7 ng/ml (from 62.3 ng/ml 14 months earlier). Histologic and immunohistochemical analyses of gastric biopsy specimen demonstrated diffuse mucosal infiltration of MTC (Fig. 1d–h) with a KI-67 index of up to 40 %, proving metastatic disease of MTC to the gastric mucosa. Of note, while immunohistochemical analysis of calcitonin was strongly positive (Fig. 1g), staining for CEA demonstrated merely focal and weak staining (Fig. 1h); staining for thyroglobulin remained completely negative (data provided in Additional file 1). She rapidly deteriorated and died due to multiorgan failure before third-line treatment could be initiated.

## Conclusions

MTC is sporadic in origin in 60 to 75 % of all patients with MTC, while the others exhibit germline mutations in the *RET* proto-oncogene, namely patients with multiple endocrine neoplasia type 2A (MEN2A), multiple endocrine neoplasia type 2B (MEN2B), and familial MTC syndrome (FMTC). The most common sites for metastatic disease in MTC are locoregional lymph nodes; these metastases often occur early in the disease process [8]. This was also the case in our patient, who initially presented with a pT2pN1b tumor stage, and who received repeated resection of cervical lymph node metastases during the following years.

However, in advanced MTC, distant metastases have been described. In one report, 74 % of patients demonstrated metastatic disease to the bone [9], other groups found small hepatic metastases in 25 % of patients with advanced MTC [10], as well as lung metastases in 35 % of patients with MTC with persistently elevated calcitonin levels after initial treatment [5]. In addition, cases of cutaneous [11, 12], brain [13], and breast [14, 15] metastases of MTC have been described.

Metastatic disease to the stomach overall is a rare incidence in all cancer types, and data in the literature are based on case reports as well as small case series [16–18]. The most common primary tumors reported to spread to the stomach are melanoma, breast, lung, and esophageal carcinoma as well as renal cell cancer [17]. On clinical examination, gastric metastases present with epigastric pain, nausea, vomiting, as well as gastrointestinal hemorrhage. Mostly solitary lesions within the gastric mucosa are observed, although multiple metastatic lesions have been reported as well [17]. To the best of our knowledge, metastatic disease to the gastric mucosa of a MTC has not yet been reported in the literature.

Our patient had MTC with a follow-up history of 25 years and presented with severe epigastric pain. Multiple lesions within her gastric mucosa were histologically proved to be a diffuse metastatic infiltration of the MTC. Of note, CT scans of her chest and abdomen revealed stable pulmonary and mediastinal lymph node metastases; however, multiple supraclavicular lesions as well as a single new intrahepatic lesion were suggestive of MTC progress. Remarkably, a sharp rise in calcitonin levels was indicative of rapid tumor progression as well, while her serum levels of CEA remained rather stable.

The current case report should raise awareness of unusual gastrointestinal metastatic disease of MTC in advanced disease states, especially with the increasing prognosis of these tumors due to molecular-targeted oncologic therapies (for example tyrosine kinase inhibitors). In treating these patients, it is important to include this generally uncommon differential diagnosis for unspecific patient complaints during follow-up.

## Abbreviations

ACTH, adrenocorticotropic hormone; AJCC, American Joint Committee on Cancer; CEA, carcinoembryonic antigen; CRH, corticotropin-releasing hormone; CT, computed tomography; FMTC, familial medullary thyroid carcinoma syndrome; MEN2, multiple endocrine neoplasia type 2; MEN2A, multiple endocrine neoplasia type 2A; MEN2B, multiple endocrine neoplasia type 2B; MTC, medullary thyroid carcinoma

## Additional file

Additional file 1: Immunohistochemical staining of gastric biopsy specimen for thyroglobulin remained completely negative. (TIF 1120 kb)
