# Supplementary figures and images for: Unusual gastric mucosal infiltration by a medullary thyroid carcinoma: a case report
Source: J Med Case Rep. 2016 Jul 27;10:208. doi: 10.1186/s13256-016-0981-9 (PMC4962496; doi:10.1186/s13256-016-0981-9)

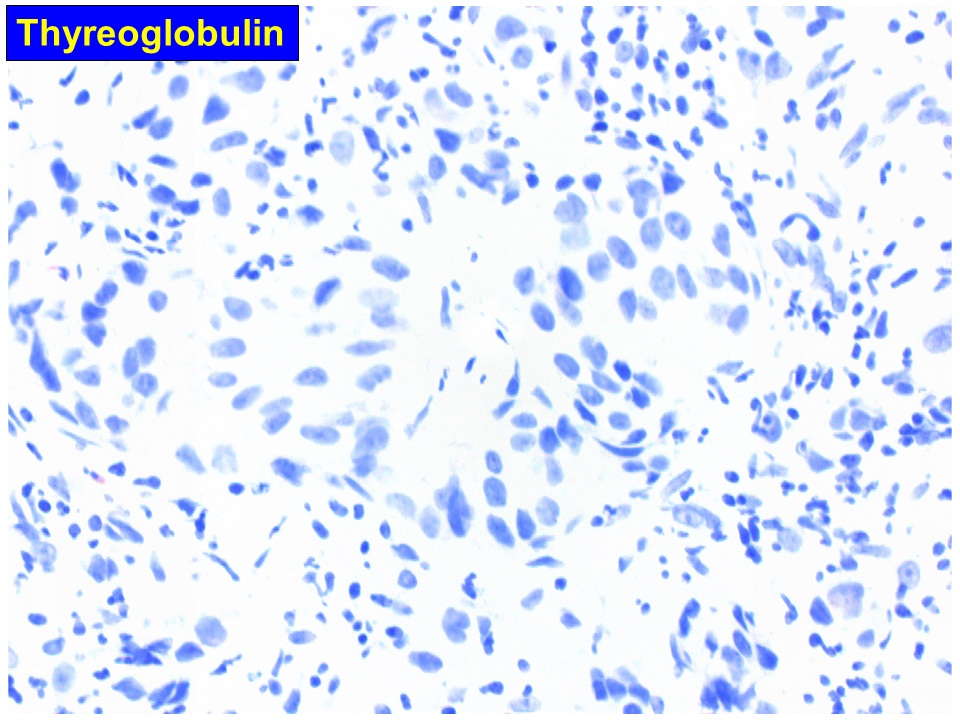

Supplement: Additional file 1: — Immunohistochemical staining of gastric biopsy specimen for thyroglobulin remained completely negative. (TIF 1120 kb) [file 13256_2016_981_MOESM1_ESM.tif]
